# Empowering informal caregivers and nurses to take a person-centred view: adaptation and clinical utility of the Integrated Palliative Outcome Scale (IPOS-Dem) for use in acute and community care settings

**DOI:** 10.1186/s12877-024-05608-8

**Published:** 2024-12-21

**Authors:** Susanne de Wolf-Linder, Iris Kramer, Margarete Reisinger, Fliss E. M. Murtagh, Maria Schubert, Christina Ramsenthaler

**Affiliations:** 1 Department of Health Sciences, Institute of Nursing, ZHAW Zurich University of Applied Sciences, Katharina-Sulzer Platz 9, CH-8400 Winterthur, Switzerland; 2 Wolfson Palliative Care Research Centre, Allam Medical Building, Hull York Medical School, University of Hull, Hull, HU6 7RX UK

**Keywords:** Dementia, Palliative care, Person-centred outcome measures, Cultural adaptation, Clinical utility

## Abstract

**Background:**

Dementia is a progressive and terminal illness. Symptoms are present for people with dementia across all stages, leading to poor quality of life and considerable carer burden. In acute and community care services, no holistic, person-centred outcome tools are available for nurses and informal caregivers to measure symptoms and needs from the person`s with dementia point of view. We therefore undertook validation (exploring semantic/conceptual equivalence, content validity, and views on clinical utility) for a measure (IPOS-Dem) in the community and acute care setting in Switzerland.

**Methods:**

This was a rigorous, multi-step, cross-sectional, multi-method study conducted with nurses and relatives caring for people with dementia in the community and acute care setting. Multiple components were aligned: 1) forward and backward translation from German to Swiss German to achieve semantic equivalence; 2) focus groups to explore clinical utility and conceptual equivalence; 3) cognitive debriefing to review content validity. An expert review was included at the end of each phase.

**Results:**

Six people from the public and 24 nurses/relatives were included. Semantic equivalence was achieved after making 14 changes to the wording of items. Participants judged the IPOS-Dem (CH) as a clinically useful intervention in the domains of appropriateness, accessibility, practicability, and acceptability for the following reasons: (1) it enables support for informal caregivers, (2) it provides an overview of the priorities of care, thus supporting symptom review, (3) it allows nurses with different qualifications to contribute critical observations, thus fostering communication and teamwork, and (4) it increases an awareness of change in symptoms throughout the disease trajectory. In the cognitive debriefing interviews, setting and respondent-dependent differences in the conceptual understanding of item descriptors were observed for 11 of 31 items.

**Conclusion:**

In this novel work, we demonstrate the newly-translated and culturally-adapted IPOS-Dem (CH) is a relevant and comprehensive measure for persons with mild to severe dementia. It can aid a generalist workforce across settings to assess palliative care-relevant symptoms and concerns.

**Supplementary Information:**

The online version contains supplementary material available at 10.1186/s12877-024-05608-8 .

## Background

Dementia is a progressive and life-limiting illness, characterised by increasing morbidity related to the illness and co-morbid conditions [1]. Demographic changes result in two to three times more people with dementia needing care provision in different settings [2–4]. Robust numbers for Switzerland are not given, however, the Swiss Federal Office of Health estimates a total of 150,000 persons with dementia with an additional 32,200 of new diagnoses each year [5]. The preferred place of care and death of people with dementia is often the home [6]. In Switzerland – contrary to other countries – a substantial number of people with dementia remain in their own home and access community care services. These are non-profit, nurse-led organisations that offer care to more than 42,000 clients with a confirmed or suspected diagnosis of dementia [5].

While symptoms and functional decline are more pronounced in advanced dementia, symptoms are present across all stages, potentially compromising quality of life and being associated with considerable carer burden [7]. Data on the prevalence and comprehensive pattern of symptoms remains scarce and is almost solely focused on nursing homes as the place of care. There, pain, hallucinations, shortness of breath, and difficulty swallowing are highly prevalent [7–11]. Pain, agitation, anxiety, and resistiveness to care have been reported as being prevalent in 40% or more of people with dementia living at home [12]. There is evidence that symptoms are not adequately assessed or managed [1].

Although several assessment tools exist to measure single symptoms (particularly pain or neurospsychiatric/behavioural symptoms), these tools lack comprehensiveness; and a person-centred and needs-based focus [13]. Both these aspects are integral to palliative care which advises the impeccable assessment and monitoring of an individual’s main symptoms and concerns to facilitate a high quality of life [14]. The Integrated Palliative Care Outcome Scale for people with dementia (IPOS-Dem) follows this palliative care perspective [15]. The IPOS-Dem measures 31 general and palliative care specific symptoms and concerns [15]. It has been evaluated as an acceptable tool in a population of nursing homes residents with complex care needs, supporting the assessment of symptoms and concerns, even by non-professional care providers [16]. A full validation study is currently being prepared. Cultural adaptations of the English IPOS-Dem to German [17], Swiss-German [18] and Swedish [19] have been provided for the nursing home setting. What is yet missing is a version of the IPOS-Dem specifically targeting the community and the acute care settings in Switzerland.

As a first step in the psychometric validation of the IPOS-Dem, this study aims to adapt the IPOS-Dem to the Swiss-German cultural context, and to explore its clinical utility and content validity in the community and acute care settings when used as a proxy assessment for people with mild to severe dementia.

## Methods

**Fig. 1:**
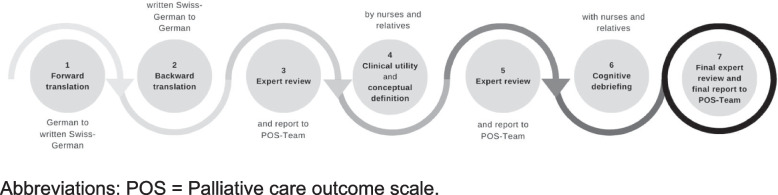
Integrated seven-step process of translation, cross-cultural adaptation, and exploration of content validity and clinical utility

This study uses a cross-sectional, explorative, qualitative multi-method design. The cultural adaptation follows guidelines by the International Society for Pharmacoeconomics and Outcomes Research (ISPOR) which comprehensively define a robust process of cultural adaptation of outcome measures [20, 21]. The guidelines focuse on the conceptual, content, semantic, and technical equivalence [22]. Since the original IPOS-Dem was developed and validated within the nursing home setting, we also explore its potential clinical utility following Smart’s model [23] in two new settings, community and acute care. Smart’s model was chosen because of its multi-dimensional and multi-perspective nature, defining clinical utility in the clinical environment, contextualising different aspects of clinical utility for patients, health care professionals, and health provision in the setting [23]. The integrated workflow over all objectives is presented in Fig. 1, with each step explained below.

### Steps 1–3: Semantic equivalence

A Swiss-German language version of the IPOS-Dem was developed from the German translation of the IPOS-Dem [17]. The German translation had been developed using the full ISPOR process. German and Swiss-German are similar languages in writing, however, the spoken languages differ in linguistic and socio-cultural factors. We therefore hypothesized that it would be feasible to work from the translated German version, but add and adapt Swiss colloquialisms in the item descriptors targeted towards the Swiss cultural context.

Instead of following the full ISPOR process of forward and backward translation [20], we used an adaptation focusing on synonyms for specific words and colloquialisms [24]. Six adult Swiss-German native speakers from the public and previously known to the researchers were purposively selected based on their distinct Swiss-German dialect, representing the dialects spoken most widely in the Swiss German part of Switzerland. They independently read the German IPOS-Dem and highlighted changes to be considered for the IPOS-Dem (CH). Discrepancies between the suggested translations were discussed with participants until agreement was reached by anonymous vote. Two researchers from nursing and ethics provided the backwards translation of the IPOS-Dem (CH) to German. They also collected as many synonyms as possible for changed words. The moment one of the synonyms matched the previous German version, this translation was used. The translated IPOS-Dem (CH) was then sent for expert review to the Palliative care outcome Scale (POS) team. Due to this phase being conducted during the COVID-19 pandemic, the entire communication was conducted online via email.

### Steps 4–5: Views on clinical utility and conceptual equivalence

In three focus group interviews with health care professionals and informal caregivers, the clinical utility [23] and the conceptual equivalence of the Swiss-German IPOS-Dem (CH) with the German [17] and the original English version [16] was explored, checking whether the same multidimensional construct of general and palliative care specific symptoms and needs for people with dementia was measured [25].

Nurse participants were purposively sampled by role, qualification, and setting (community and acute care). A convenience sample of informal caregivers, defined as family and friends of people with dementia, was recruited through existing contacts from our patient and public advisory group. A semi-structured topic guide centred around Smart’s [23] dimensions of clinical utility (appropriateness, accessibility, practicability, and acceptability) was developed for the focus groups of nurses and adapted for the focus groups with informal caregivers (Additional file 1). Prior to the focus group, each participant completed an IPOS-Dem (CH) for a person with dementia currently in their care. Focus group discussions were held online, recorded, and transcribed verbatim. Thematic content analysis in Max Weber Qualitative Data Analysis (MAXQDA) [26], following Kuckartz [27], used a deductive-inductive approach with initial categories from Smart’s model [23]. To maintain rigour in the analysis, two researchers independently coded 50% of the transcripts with discrepancies being resolved by consensus [28]. Formal expert review was sought by the POS scientific committee before proceeding to cognitive debriefing.

### Steps 6–7: Content validity

Cognitive debriefing interviews [22, 29] to assess content and technical equivalence of the IPOS-Dem (CH) were conducted with a new purposive sample of nurses (by role, qualification, and setting) and a convenience sample of informal caregivers, which was recruited through snowballing from our patient and public advisory group. Cognitive interviews were facilitated by a topic guide (Additional file 2). These interviews were conducted online (due to restrictions during the COVID-19 pandemic) in rounds of three participants until data saturation was reached [29, 30]. Participants were asked to think aloud while completing an IPOS-Dem (CH) and voicing aspects of the concepts, the relevance and their comprehension. Probing questions (prompts) were based on Tourangeau’s model of comprehension, retrieval, judgement, and response [31, 32]. Cognitive interviews were directly analysed from audio/video recordings by two independent researchers per interview. A thematic content analysis, using a deductive approach with categories from Tourangeau’s model [32], was used. Discrepancies were resolved by consensus. Changes to the IPOS-Dem (CH) were made after each round with further interviews then using the refined version of the IPOS-Dem (CH). The final version of the IPOS-Dem (CH) was sent for approval by the POS team.

## Results

Overall, 32 participants (*n* = 8 native speakers from the public, *n* = 16 nurses and informal caregivers in three focus groups, and *n* = 8 nurses and informal caregivers in the cognitive interviews, see Table 1 for demographic details) took part in the study between June 2020 and May 2021. All informal caregivers provided consent, but one failed to take part due to ad-hoc commitments. Three of 14 nurses approached had to cancel participation due to urgent patient care.

**Table 1:** Demographic characteristics for all parts of the study and all samples

Steps	Role (Participant)		Gender		Setting	
***1–3: Semantic equivalence***	Dialect Valais	1	Women	5	Citizen	6
	Dialect Zurich	1	Men	3	Research institute	2
	Dialect Glarus	2				
	Dialect Bern	1				
	Dialect Lucerne	1				
	Nurse researcher	1				
	Ethics researcher	1				
	**Total**	**8**				
***4–5: Clinical utility, content and conceptual equivalence***	Relative – daughter	1	Women	12	Home (family)	4
	Relative – son	1	Men	4	Acute care	6
	Relative – husband	2			Community care	5
	Nurse – university degree	9				
	Nurse – professional degree	2				
	**Total**	**16**				
***6–7: Content validity***	Relative – daughter	2	Women	7	Home (family)	2
	Nurse – university degree	3	Men	1	Acute care	3
	Nurse – professional degree	3			Community care	3
	**Total**	**8**				

All interviews lasted between 60 and 80 min. Participants in the focus groups took a mean of seven minutes to complete the IPOS-Dem (CH).

### Steps 1–3: Semantic equivalence

In the forward translation, 14 changes in wording were made to the 31 items of the IPOS-Dem (CH) (e.g., from I9. *Does not enjoy eating* to I9. *Does not feel like eating*). The most discussed items, therefore presenting a challenge in translation, were I22. *Wandering*, I16. *Artificial dentures (problems with teeth)*, and I18. *Skin breakdown*. A considerable change was proposed for I31. *Has the person had any other symptoms? Please select one box to show how you feel each of these symptoms have affected the person over the past week (optional).* In the forward translation, it was suggested to extend the item text to encompass physical, emotional, social, or spiritual symptoms. This longer version was confirmed via the backward translation. The POS team approved the IPOS-Dem (CH) translation.

### Steps 4–5: Clinical utility and conceptual equivalence

#### Step 4: Clinical utility

Four dimensions from Smart’s [23] model of clinical utility were used to integrate the 12 inductive themes from the focus groups (Table 2).

**Table 2:** Four dimensions and selected aspects for exploring the potential clinical utility of the IPOS-Dem (CH) in acute and community care settings

**Dimensions** (Smart [23])	**Selected aspects for the focus group discussions**	**Results: corresponding themes**
Appropriate	• Impact of IPOS-Dem (CH) on existing treatment processes (Is the IPOS-Dem effective?) • Importance of clinical decision-making when using the IPOS-Dem (CH) (Is it relevant?)	• IPOS-Dem (CH) enables better support of the person with dementia • Clinical decisions are based on symptoms important to people with dementia and their informal caregivers (to fine-tune care, particularly for informal caregivers) • Guidelines needed for the clinical interpretation of scores (nurses) • Improved awareness of change in health status and disease severity (informal caregivers)
Accessible	• Economic considerations, e.g., procurement including availability of technologies, quality of material, availability of training and support	• IPOS-Dem (CH) to be assessed, saved and documented using an online system (community nurses) • Being able to access the IPOS-Dem symptom profile/changes in the symptom profile on a computer (community nurses)
Practicable	• Functionality within work context • Suitability (e.g., is it helpful in everyday situations) • Practitioner skills and capabilities (e.g., professional qualifications)	• IPOS-Dem (CH) to support communication and teamwork (acute care nurses) • IPOS-Dem (CH) captures observations from nurses with different qualification levels, therefore valuing all members of the care team equally (all settings) • IPOS-Dem (CH) provides an overview of the priorities of care, therefore helps with communication to the community care provider (informal caregivers) • IPOS-Dem (CH) may support symptom review (community nurses)
Acceptable	• Possible moral objections when administering the IPOS-Dem (CH) (e.g., beneficial to the person with dementia and informal caregivers) • Clarification of expectations regarding service delivery and person with dementia	• IPOS-Dem (CH) giving an objective account of symptoms and change in health status (informal caregivers, community nurses) • Completing an IPOS-Dem (CH) in the acute setting can be a challenge if one does not know the person with dementia well (acute care nurses)

##### Appropriateness

Effectiveness and relevance as aspects of appropriateness were evidenced by statements in the focus groups of both nurses and informal caregivers. Both groups valued the IPOS-Dem (CH) as a person-centred outcome measure for integration into everyday care and across the whole care continuum.

All participants regarded the IPOS-Dem (CH) as effective in providing a reliable, short and person-centred assessment of symptoms. However, while nurses wanted to base clinical decisions on the outcome measure, they also raised concerns about the interpretation of scores. They missed clear guidance regarding the level of scores that would indicate the need for clinical intervention. Contrarily, informal caregivers felt empowered to fine-tune care to individual symptoms that had been scored high without needing a cut-off score. This group particularly valued the ability to recognise change or a deteriorating health status earlier: *“Having had the IPOS-Dem (CH) at hand would have supported me in organising support earlier – also support from the outside, which was much needed.”* (*P2, son*).

##### Accessibility

Touching on economic aspects and aspects of accessibility, community care nurses asked for an electronic version of the IPOS-Dem (CH), currently only available in paper format, for integration into electronic documentation systems. They suggested to include a longitudinal symptom profile for the easy monitoring of changes. An electronic format could help justifying benefit claims and insurance payments: *“I think it is an important tool in addition to regular evaluations to underpin and stress the needs of the person with dementia, which would help to justify benefit approvals, rejections from the insurance and/or health insurance” (Nurse 5, community care).*

##### Practicability

In the acute and community care setting, IPOS-Dem (CH) can support communication during handovers. Handovers would become more structured, thereby streamlining communication for the whole team. Individual nurses less familiar with the patient would gain an instant overview of the symptom profile. The IPOS-Dem (CH) was appraised as appropriate for eliciting priorities of care, therefore saving time in both acute and community care settings. Since all healthcare providers and even minimally trained health care assistants can use the IPOS-Dem (CH), its implementation can strengthen their contribution to the care of the person with dementia. This asset was particularly valued in the community care settings where many more providers with different roles and qualifications are involved.

Informal caregivers felt that the IPOS-Dem with repeated measurement could provide evidence of a changed situation when talking to health care professionals: *“At the time she was at home there was a monthly change in her symptoms and needs. With the IPOS-Dem one could have seen the different scores. I could have gained an overview and I would have been able to explain my concerns much better to healthcare professionals.” (P1, husband).*

##### Acceptability

Community care nurses and informal caregivers found the IPOS-Dem (CH) to be acceptable, particularly in the context of giving an objective account of symptoms and a possible change in health status by feature of its clearly stated and targeted items. Informal caregivers wished for the IPOS-Dem (CH) to be introduced early in the care of their loved ones to have this objective account and to be able to monitor changes.

In the acute care setting, however, the IPOS-Dem (CH) posed a challenge due to its person-centred nature. Not knowing the person with dementia well led to questioning the accuracy of scores. Completion would also require a team effort: *“In our daily surgical ward routine, we would have to complete the IPOS-Dem for the patient as a team. One person alone would not have enough information nor does the information from the electronic documentation suffice.” (Nurse 4, acute care).*

### Step 5: Conceptual equivalence

The symptoms and issues addressed in the IPOS-Dem (CH) were thought to reflect the construct of general and palliative care specific symptoms and needs of people with dementia. However, both respondent groups asked for “physical touch” to be added to the item descriptor for I29. *Have you been able to interact with others (including through physical touch), e.g. with professionals, family, others* to capture this means of communication (e.g. for feeling body tension or reaction). I12 was changed from *Drowsiness (sleepiness during the day)* to *Sleepiness (during the day)*, following comments from nurses advising the potential ambiguity of two descriptors and drowsiness not being considered a symptom or need in dementia.

I21. *Agitation* sparked an in-depth discussion in the focus group of nurses. Agitation was understood as meaning restlessness. However, in the translation process restlessness was also suggested as a synonym for I23. *Feeling anxious*. Contrarily, relatives felt that anxiety and insecurity were much closer related conceptually than anxiety and restlessness. They perceived restlessness more as a consequence of feeling anxious.

### Steps 6–7 Content validity

A total of eight interviews were conducted in three rounds. The third round involved two interviews with acute care nurses to confirm the need for setting-specific versions of the IPOS-Dem (CH), necessitated by differences in comprehension, judgement, and response to items amongst acute care and community care nurses. *Technical equivalence* of both versions was achieved by streamlining the introductory text and answer options.

Overall, cognitive interviews elicited changes made to 11 items. The following changes were made to items in both versions (acute and community care setting): I11. *Dry or sore mouth* (”sore” instead of “painful”), I19. *Difficulty communicating* (deletion of item descriptors “through speech or body language”), I22. *Wandering (as a sign of despair* (changes made to the item descriptor) I28. *Do you think that s/he felt at peace? (if the person with dementia is not well known to you, please actively seek perspectives from family and friends)* (adding the explanation for easier scoring).

Version-specific changes were made to seven items. These targeted the item descriptors or giving further examples and were due to comprehension issues (see Table 3). Changes made to items I1 and I3 (main problems) focused on clarifying the person-centered perspective. For item I13. *Limited mobility,* the addendum of “walking aids” was seen as critical in both settings. Informal caregivers perceived the use of walking aids to be normal, therefore not indicating a true change in mobility for that person. Nurses from the acute care setting adviced to include a wider range of mobility, e.g. being able to change the position in bed. The original I13 also had the item descriptor of “falling”. Both nurses and informal caregivers proposed deleting this descriptor since other reasons could lead to falls in dementia (construct contamination). Likewise, “lying in bed” was challenged as an item descriptor for I13:

*“The explanation “cannot get out of bed” may not relate to limited mobility because sometimes people with dementia just don’t want to get up. Maybe it is better to state ‘cannot climb stairs easily’.” (Round 1: relative)*

**Table 3:** Overview of setting-specific item changes for the community care and acute care versions of the IPOS-Dem (CH) after each round of cognitive debriefing

IPOS-Dem (CH) item after steps 1–3: Translation and semantic equivalence	Comments from community care nurses	Comments from acute care nurses	Comments from informal caregivers	Changes to the item of the IPOS-Dem (CH) Community nurses and community care setting or IPOS-Dem CH acute care setting
Explanatory text before completing each item: (…) Try to imagine how the person with dementia experiences the current situation. (…)	1* The IPOS-Dem takes the view from the person with dementia, “…try to imagine…” does not pay justice	1 no changes proposed	1 no changes proposed	(…) Try **to consider** how the person with dementia (…)
	2* no changes proposed	2 no changes proposed	2 no changes proposed	
	3* no changes proposed	3 no changes proposed	3 no changes proposed	Version for community care and acute care setting the same: (…) Try to consider how the person with dementia experiences the current situation. (…)
Answer option: Cannot assess (e.g., unconscious)	1 no changes proposed	1 no changes proposed	1 no changes proposed	
		2 “Unconscious” might be difficult for relatives to comprehend	2 Difficult to comprehend “unconsciousness” in relation to the items; to be deleted	Answer option: Version community care: Cannot assess without further information in brackets
	3 no changes proposed	3 no changes proposed	3 no changes proposed	Answer option amended in the version community care and version acute care: Answer option: Cannot assess
I1. What have been the person’s main problems over the course of the last week?	1 no changes proposed	1 no changes proposed	1 no changes proposed	
	2 no changes proposed	2 no changes proposed	2 no changes proposed	
	3 no changes proposed	3 unclear at whom this item is aimed, to add “from their point of view”	3 no changes proposed	Version community care: no amendments Version acute care: What have been the person’s main problems over the course of the last week from **their point of view**?
*I2. What have been the person’s family member’s main concerns over the course of the last week?*	NA**			*What have been the person’s family member’s main concerns over the course of the last week?*
I3. What have been the main problems in the care and provision of the person with dementia over the course of the last week?	1 No changes proposed	1 Change from “problems” to “challenges”	1 Who is supposed to answer this item? To whom is it addressed?	What have been the main **concerns** in the care and provision of the person with dementia over the course of the last week?** (If the person with dementia is not well known to you, please actively seek perspectives from family and friends)**
	2 no changes proposed	2 Perspective in terms of the setting still unclear	2 “Care” relates to community care services and provision relates more to food, shelter, etc	What have been the main concerns in the **care or care provision at home / in hospital** of the person with dementia over the course of the last week? (If the person with dementia is not well known to you, please actively seek perspectives from family and friends)
	3 no changes proposed	3 Unclear which perspective to take based on information in brackets	3 no changes proposed	Version community care: What have been the main concerns in the care **and/**or care provision at home / in hospital of the person with dementia over the course of the last week? (If the person with dementia is not well known to you, please actively seek perspectives from family and friends) Version acute care: What have been the main concerns in the **professional** care and / or care provision of the person with dementia over the course of the last week?
*I4. Pain*	*NA*			*Pain*
*I5. Shortness of breath*	*NA*			*Shortness of breath*
*I6. Weakness or lack of energy*	*NA*			*Weakness or lack of energy*
*I7. Nausea (feeling like being sick/vomiting)*	*NA*			*Nausea (feeling like being sick/vomiting)*
*I8. Vomiting (being sick)*	*NA*			*Vomiting (being sick)*
*I9. Does not feel like eating* *(e.g., does not eat when prompted, pushes food away)*	*NA*			*Does not feel like eating* *(e.g., does not eat when prompted, pushes food away)*
*I10. Constipation*	*NA*			*Constipation*
I11. Dry mouth or pain in the mouth	1 no changes proposed	1 Unsure how the item relates to other items about pain, dentures and practical problems	1 no changes proposed	The word “painful” replaced with “sore” to avoid confusion: Dry or sore mouth
	2 no changes proposed	2 no changes proposed	2 no changes proposed	
	3 no changes proposed	3 no changes proposed	3 no changes proposed	Version for community care and acute care setting the same: Dry or sore mouth
*I12. Sleepiness (during the day)*	*NA*			*Sleepiness (during the day)*
I13. Limited mobility (e.g., trouble walking, cannot leave bed, falling)	1 If the person with dementia is using a Zimmer frame, that would indicate that they are fully mobile	1 Explanations in brackets are of importance for nurses	1 “cannot get out of bed” does not accurately reflect the dementia population	Limited mobility (e.g., trouble walking, **cannot climb stairs**, falling)
	2 no changes proposed	2 Range of mobility from walking to change of position in bed	2 Item should ask about mobility including walking aids	Limited mobility (e.g., trouble walking, cannot climb stairs, **trouble changing position**, falling)
	3 “Falling” is understood as a risk/screening question rather than belonging to limited mobility	3 Importance of information in brackets emphasized – in particular “trouble changing position”	3 Falls may be understood as a separate event	“Falling” excluded from information in brackets Version for community care: Limited mobility (e.g., trouble walking, cannot climb stairs) Version acute care: Limited mobility (e.g., trouble walking, cannot climb stairs, trouble changing position)
I14. Sleeping problems (at night)	1 no changes proposed	1 no changes proposed	1 no changes proposed	
	2 no changes proposed	2 Information in brackets “(at night)” is not needed because of the frequently observed sundowning syndrome in people with dementia	2 no changes proposed	Version community care: Sleeping problems (at night) Version acute care: Sleeping problems
	3 no changes proposed	3 no changes proposed	3 no changes proposed	
*I15. Diarrhoea*	*NA*			*Diarrhoea*
*I16. Dental problems or problems with artificial dentures*	*NA*			*Dental problems or problems with problems with artificial dentures*
*I17. Swallowing problems (e.g., chokes, inhales food or drink, holds food in mouth)*	*NA*			*Swallowing problems (e.g., chokes, inhales food or drink, holds food in mouth)*
*I18. Skin breakdown (e.g., cracked skin, pressure sores, itching/biting)*	*NA*			*Skin breakdown (e.g., cracked skin, pressure sores, itching/biting)*
I19. Difficulty communicating (through speech or body language)	1 no changes proposed	1 no changes proposed	1 no changes proposed	
	2 no changes proposed	2 no changes proposed	2 Important item but information in brackets can confuse if not applicable	Delete descriptors in brackets Difficulty communicating
	3 no changes proposed	3 Item as is includes all forms of communication	3 no changes proposed	Version for community care and acute care setting the same: Difficulty communicating
I20. Hallucinations (seeing or hearing things that are not present) and/or delusions (to belief in something that is not real)	1 “Delusion” is a negatively connotated word which should not be used for people with dementia as it is often associated with “madness”	1 Hallucinations and delusions are not the same symptom. Hallucinations are difficult to assess	1 People with dementia are often referring to the past. These are not hallucinations	Deleting the medical terms, keeping the item descriptor: Seeing or hearing things that are not present and/or believing something that is not real
	2 Item can also indicate a delirium	2 Maybe include “smelling” into descriptor	2 The word “present” to be exchanged with “possible” as the latter is more commonly used in the Swiss German language	Version community care: Seeing or hearing things that are not **possible** and/or believing something that is not real Version acute care: Seeing, hearing, **or smelling** things that are not **possible** and/or believing something that is not real
	3 no changes proposed	3 no changes proposed	3 no changes proposed	
I21. Agitation (restless)	1 If someone is agitated, then the person becomes more restless	1 Agitation and restlessness are the same concept	1 Agitation is nestling; restlessness is walking around. If people with dementia feel threatened, they become restless	Agitation (restlessness **as a consequence of feeling distressed**)
	2 Restlessness is more a long-term condition. Agitation is often caused by something acute and is therefore a reason for concern	2 Agitation means that the person with dementia is driven by something	2 Restlessness is the smaller version of agitation	Round 1 alteration taken out because restlessness was not clearly related to feelings of angst: Agitation (restlessness)
	3 Two different terms within the same concept	3 Restless means pulling out drips/tubes, getting out of bed, verbal aggressiveness	3 no changes proposed	Version community care: Agitation (restlessness) Version acute care setting: Agitation
I22. Wandering (as a result of restlessness or feeling threatened)	1 The item is connected to I21	1 Maybe this is a sub-category of the previous item? (Agitation (restlessness))	1 Feeling threatened results more in agitation than wandering. Wandering is a complex phenomenon in people with dementia	Expert consultation – change descriptor in brackets since wandering is not necessarily a result of restlessness: Wandering (**e.g., as a sign of distress/despair**)
	2 no changes proposed	2 Only asks about wandering if person with dementia is distressed	2 “Sign of distress” might be too narrow for this complex symptom, also narrows the symptom to distress	Wandering (as a sign of **despair**)
	3 Wandering in despair is different to restlessness but it also complements the two items	3 Adding despair as a descriptor makes it easier to understand	3 no changes proposed	Version for community care and acute care setting the same: Wandering (as a sign of despair)
*I23. Has s/he been feeling anxious or worried?*	*NA*			*Has s/he been feeling anxious or worried?*
*I24. Have any of his/her family been anxious or worried about the person?*	*NA*			*Have any of his/her family been anxious or worried about the person?*
*I25. Do you think the person with dementia felt depressed?*	*NA*			*Do you think the person with dementia felt depressed?*
*I26. Did s/he lose interest in things that s/he would normally enjoys?*	*NA*			*Did s/he lose interest in things that s/he would normally enjoys?*
*I27. Has s/he been showing irritated or aggressive behaviour?*	*NA*			*Has s/he been showing irritated or aggressive behaviour?*
I28. Do you think s/he felt at peace?	1 no changes proposed	1 no changes proposed	1 no changes proposed	
	2 no changes proposed	2 no changes proposed	2 Able to complete this item easily for her mum (daughter), no problems with comprehension or assessment	No changes
	3 This item might reflect truly the quality of our care because it might indicate how well the person with dementia is supported by us	3 Item can only be answered based on long conversations with the person with dementia or their family	3 no changes proposed	Information added for nurses who have not known the person with dementia over a longer period of time Version for community care and acute care setting the same: Do you think s/he felt at peace? **(If the person with dementia is not well known to you, please actively seek perspectives from family and friends.)**
I29. Has s/he been able to interact with others (including physical contact e.g., with staff, family, residents)?	1 no changes proposed	1 no changes proposed	1 no changes proposed	
	2 no changes proposed	2 no changes proposed	2 no changes proposed	
	3 no changes proposed	3 in hospital we would use the term patients and not residents		Version community care: Has s/he been able to interact with others (including physical contact e.g., with staff, family, residents)? Version acute care: Has s/he been able to interact with others (including physical contact e.g., with staff, family, **patients**)?
I30. Have any practical problems been addressed? (e.g., hearing aids, foot care, glasses, diet)	1 no changes proposed	1 no changes proposed	1 no changes proposed	
	2 no changes proposed	2 no changes proposed	2 no changes proposed	
	3 no changes proposed	3 Unclear what is meant by diet. Item needs to be clearer (content and perspective of observer)	3 no changes proposed	Version community care remained the same: Have any practical problems been addressed? (e.g., hearing aids, foot care, glasses, diet) Version acute care setting: Have any practical problems addressed? (e.g., **supportive devices addressed**, hearing aids, foot care, glasses)
*I31. Other physical, emotional, social, or spiritual symptoms*	*NA*			*Other physical, emotional, social, or spiritual symptoms*

^*^1, 2, 3 indicate round 1, round 2, round 3 of cognitive interviews

^**^NA = not applicable

Problems with I21. *Agitation* have already been described above. Acute care nurses felt the item descriptor I14. *Sleeping problems* (at night) not relevant because many admitted people with dementia would present with a reversed day/night routine (sundowning syndrome). Regarding I20. *Hallucination and delusion*, not all participants understood the correct meaning of the medical terms hallucinations and delusions:

*“People with dementia see things that were there in the past and which are voiced now. This is not a hallucination” (Round 1: relative)*

Hence, the medical terms were deleted and the formerly used item descriptors *Seeing or hearing things that are not there and/or believing something that is not real* were used as the new item text. It was important for acute care nurses to add *smelling* to the item text. In consecutive interview rounds, the item was interpreted by respondents as still asking about hallucinations or delusions. No further issues regarding comprehension, retrieval, judgement, and response were raised. All changes were approved by the POS development team (for final versions please see Additional file 3 and 4).

## Discussion

Our study shows that the IPOS-Dem (CH) in its cultural adaptation to the Swiss context and two new settings – the acute care & community care setting – is a relevant and comprehensive measure for people with dementia. We demonstrate that despite its length, it is an easy-to-use and short measure that can be integrated into everyday clinical practice, capturing a comprehensive set of symptoms and concerns that transcends commonly used domain-specific measures in dementia care (e.g., tools such as the PAINAD for assessing pain, or the Neuropsychiatric Interview for assessing neuro-behavioural problems). In the translation and adaptation, we primarily exchanged or added to the item descriptors but kept the initial item text, thereby ensuring content validity and improved comprehension and consistency in interpreting items by all assessors – healthcare professionals, assistants and informal caregivers. Therefore, we considerably extend the initial development and process evaluation [15, 16] to these new settings, also ensuring that all people involved, regardless of their training status or specialist knowledge, can contribute to fostering a person-centred perspective in dementia care. With many more people living with dementia worldwide, these results are important to combat the risk of symptoms being under-detected and under-treated.

We chose to start the adaptation process not from the original UK but the German version [17]. The German version had been robustly adapted from the English original, however, despite the similarities in written German and Swiss German, various spoken Swiss German dialects and different dementia care structures within the Swiss healthcare system necessitated a translation to the Swiss context. Unlike other development and translation studies, the authors also used a mixed professional/non-professional sample. Two items from the original UK version had already been removed from the German version (item “Enjoying activities appropriate for their level of interests and abilities” and item “Family having had as much information as wanted”). Both items had shown comprehension, retrieval and response problems in other studies [15, 19] and were deemed appropriate for the long-term institutional but not the community setting, a result replicated in our study.

Items consistently posing a challenge for conceptual equivalence and content validity throughout all phases of our study were I14. Sleeping problems (item descriptor not needed in the acute care setting), I20. Hallucinations/delusions, I21. Agitation (restless), I22. Wandering (changed item descriptor to “as a sign of (distress/) despair”), I29. Interacting with others (adding physical touch as a means of communication), mentioned by informal caregivers as an effective facilitator in communicatively impaired advanced dementia [33], and I30. Practical problems. Comprehension or adaptation issues with these items have been unanimously identified in prior studies [15, 17–19]. In I20, the use of the technical medical terms hallucinations and delusions necessitated an adaptation in the item descriptors for universal understanding by all respondent groups. Martinsson & Sahlén [19] also noted that hallucinations/delusions may transport a negative connotation that could lead to stigmatisation. In our study, the item descriptors emerged as a crucial addition for non-medically trained assessors. I21. Agitation had been split into two items in the German translation [17], due to concept contamination between agitation/restlessness and irritability/aggression. The authors decided to keep restlessness as an item descriptor for agitation. This empirical result was replicated in our study, thereby rejecting the consensual concept definition of the International Psychogeriatrics Association’s Agitation Definition Working Group [34] which explicitly includes emotional distress and manifest aggressive behaviour as elements of agitation. Again, setting-specific differences in the semantic understanding were voiced by acute care nurses, the only respondent group that followed this definition. Contrarily, community care nurses judged the item descriptors as too narrow an attribution for agitation. Similar problems were seen for I22. Wandering. A different understanding of behavioural symptoms in dementia could either be explained by the different challenges in the respective environments (e.g., time available, care processes) or by differences in staff training [35]. Also, acute care nurses may see people with dementia often in an agitated state when admitted to hospital [36], thus interpreting it as a sign of dementia rather than as an expression of distress/anxiety or despair.

These findings demonstrate that, firstly, using minimally trained proxies to assess palliative care related symptoms and concerns in dementia is feasible, with fewer comprehension, retrieval, judgment, and response problems reported than in comparable studies. Secondly, the use of all people involved in the care of the person affected by dementia can foster and deepen a person-centred perspective even in advanced stages or in settings where professional staff (due to turnover, care processes, or communication difficulties) experiences problems of judgment [37]. Evidence from specialist palliative care supports the shift to person-centred care planning after the systematic introduction of outcome measures [38–41], also combatting an overly task-based care culture [42]. Thirdly, the provision of additional and clear item descriptors is important to enable non-trained or minimally trained proxies to assess symptoms and concerns, a finding in stark contrast to other outcome measures that exclusively target professional assessors [13].

**Fig. 2:**
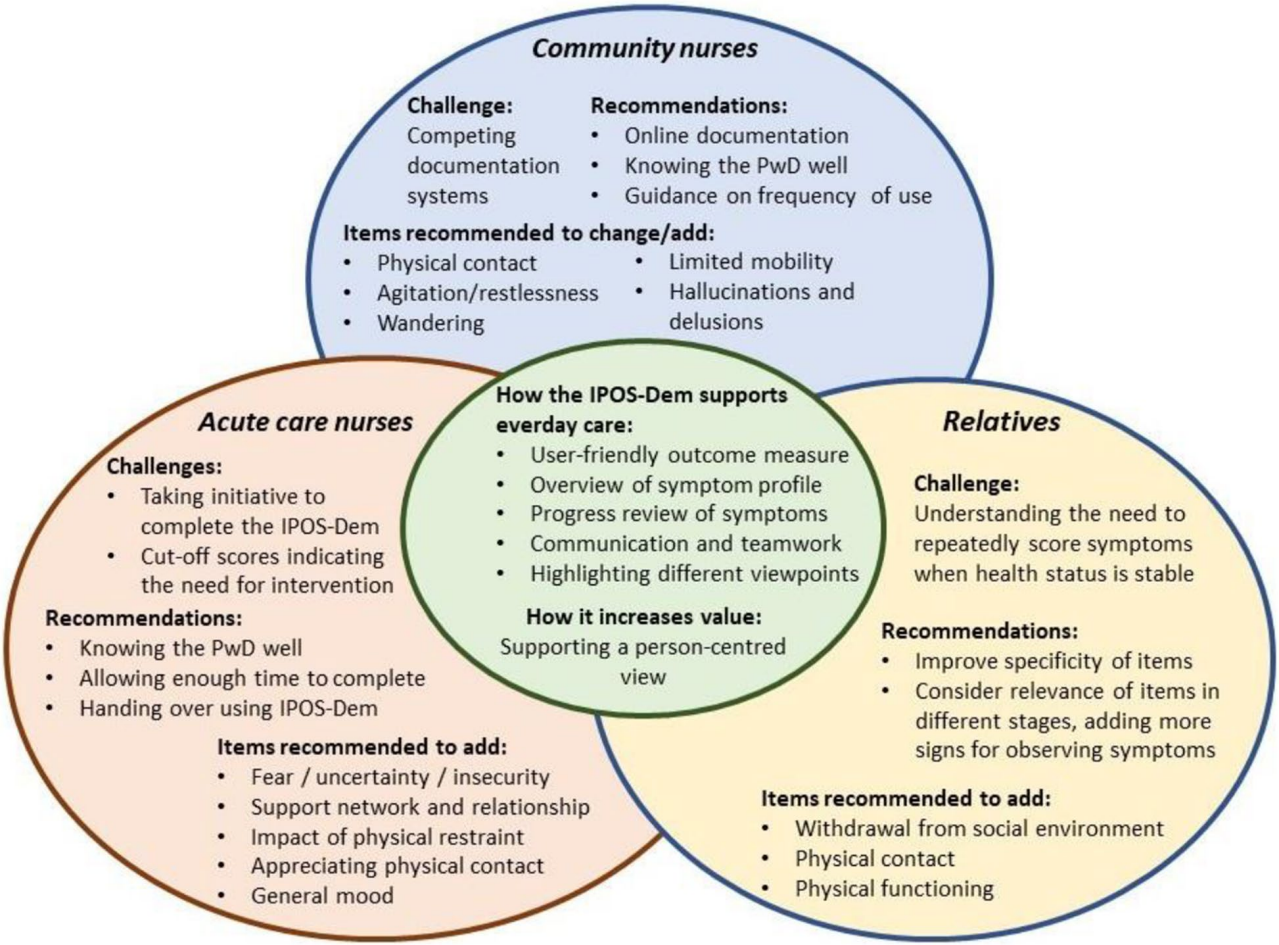
Integration of findings regarding the equivalence, content validity and clinical utility of the IPOS-Dem (CH)

Findings from our semantic equivalence, content validity, and clinical utility are triangulated over settings and perspectives (see Fig. 2). The IPOS-Dem (CH) was regarded as strengthening the provision of high-quality care through communication and teamwork, both through the inclusion of multiple perspectives and the necessity to collaboratively assess persons with dementia not well known to the team [43]. Respondents from our study voiced aspects of the potential clinical utility of outcome measures being used as complex interventions in routine care. An improved observation and collaborative assessment of the person with dementia, resulting in a holistic, person-centred and systematic monitoring which enabled care planning and care provision have been demonstrated as key mechanisms of action in a process evaluation of the IPOS-Dem in dementia care [16]. Furthermore, clear benefits for informal caregivers with increased empowerment and engagement in care as well as an improved communication between providers have also been recognised [16, 38, 44, 45]. Potential challenges to a successful implementation of the IPOS-Dem (CH) into everyday care also mimic those identified in these reviews and, mainly referring to the issue of competing, non-person-centred assessments and the lacking electronic readiness of institutions, as well as the necessity of good leadership, organisational drive, peer support, and training.

## Limitations

Our work builds on the translated and culturally adapted German version of the IPOS-Dem [17]. Thus, the conceptual equivalence may be jeopardised due to potential misconceptions being included in the German version without intent. However, Hodiamont et al*.* [17] captured rich data from all respondent groups (nurses, nursing assistants, informal caregivers). Quality assurance was provided by the POS-Team after each step. The second limitation is the online format of the focus group interviews due to the COVID-19 pandemic. This might have prevented richness of data and distraction from the interview due to technical aspects. However, the online format allowed achieving data saturation during the pandemic. Due to the COVID-19 pandemic, it was not possible to recruit participants in person and we therefore used existing contacts through our patient and public advisory board. This might have negatively affected the representativeness of the purposively drawn sample. Four out of six informal caregivers were daughters and sons, implying a selection bias because most people with dementia live with their spouses [48]. Potential spouse participants may have declined participation due to the absence of alternative caregivers or to competing demands related to their health conditions and responsibilities [48]. Finally, our study used an explorative, qualitative design to investigate the potential clinical utility of the IPOS-Dem after a one-off use. Further studies need to investigate the effects regarding its clinical utility after routine implementation.

## Conclusion

The further development of dementia-specific outcome measures has been identified as a main research priority since the last decade [46]. This is the first study to demonstrate the content and face validity of the IPOS-Dem, a comprehensive, proxy-reported, person-centred outcome measure of general and palliative care related symptoms and concerns in dementia, in two new settings, the acute and the community care setting, and across a wide range of respondent groups. This extends the original development and further work in the long-term care setting [15–19]. Clarifying conceptual ambiguities and contrasting different understanding of items and item descriptors further helps to define the construct to be measured and thus helps prepare the full validation study. The IPOS-Dem (CH) may aid a generalist workforce across settings to assess palliative care relevant symptoms and concerns.

## Supplementary Information

Additional file 1. Topic guide for focus groups (views on clinical utility and conceptual equivalence).

Additional file 2. Topic guide for cognitive interviews (views on content validity).

Additional file 3. IPOS-Dem (CH) and back-translated English version for the community care setting.

Additional file 4. IPOS-Dem (CH) and back-translated English version for the acute care setting.

## Data Availability

All data generated or analysed during this study are included in this published article [and its supplementary information files].

## Abbreviations

ISPOR: International Society for Pharmacoeconomics and Outcomes Research
IPOS-Dem (CH): Integrated Palliative Care Outcome Scale for Dementia Switzerland
MAXQDA: Max Weber Qualitative Data Analysis
POS: Palliative Care Outcome Scale
