# Supplementary material for: Empowering informal caregivers and nurses to take a person-centred view: adaptation and clinical utility of the Integrated Palliative Outcome Scale (IPOS-Dem) for use in acute and community care settings
Source: BMC Geriatr. 2024 Dec 21;24:1030. doi: 10.1186/s12877-024-05608-8 (PMC11662603; doi:10.1186/s12877-024-05608-8)
Supplement: Supplementary file 1 — Additional file 1. Topic guide for focus groups (views on clinical utility and conceptual equivalence). [file 12877_2024_5608_MOESM1_ESM.docx]

**Additional File 1 to «Empowering carers and nurses to take a person-centred view: Adaptation and clinical utility of the Integrated Palliative Outcome Scale (IPOS-Dem) for use in acute and community care settings»**

Topic guide for focus groups (views on clinical utility and conceptual equivalence)

Participants: Nurses and informal carers

Setting: Online

Duration: max. 60 minutes

1. Introduction of study team and participants
2. Clarification of any questions related to the interview or study before proceeding
3. Oral re-consent from participants
4. Completing the IPOS-Dem: “*I would like to ask you to complete the IPOS-Dem (CH) now from the perspective of your relative / client / person with dementia you have most recently cared for”.*
5. Start interview and record:

- Question 1: How do you think this questionnaire (IPOS-Dem) could support you in your everyday life or nursing routine?

Prompts: what are the pros and cons? What would increase the value of the IPOS-Dem in your everyday life / nursing routine? Refer to previous focus-groups in focus group interview two and three if applicable.

- Question 2: From your point of view, is something missing in the IPOS-Dem?
- Which questions could be left out?
- Possible scenarios: If we add additional questions than it would become longer, do you think we asked the right questions. Could we leave an item out if we add additional questions?
- Question 3: How do you interpret the answer options?
  Prompts: Depending on the individual answer, how would you act upon and when and why? Is the choice of answer options reasonable from your point of view? If yes / no, why?
- Question 4: Is there anything else you would like to add?

1. Stop recording.
2. Thank you to participants / offer support telephone number of study team should any issues arise after the interview.
