# Supplementary material for: Empowering informal caregivers and nurses to take a person-centred view: adaptation and clinical utility of the Integrated Palliative Outcome Scale (IPOS-Dem) for use in acute and community care settings
Source: BMC Geriatr. 2024 Dec 21;24:1030. doi: 10.1186/s12877-024-05608-8 (PMC11662603; doi:10.1186/s12877-024-05608-8)
Supplement: Supplementary file 2 — Additional file 2. Topic guide for cognitive interviews (views on content validity). [file 12877_2024_5608_MOESM2_ESM.docx]

**Additional file 2** **to «Empowering carers and nurses to take a person-centred view: Adaptation and clinical utility of the Integrated Palliative Outcome Scale (IPOS-Dem) for use in acute and community care settings”**

Topic guide for cognitive interviews (views on content validity)

Participants: Nurses and informal carers

Setting: Online

Duration: max. 60 minutes

1. Introduction of researcher and participant
2. Clarification of any questions related to the interview or study before proceeding.
3. Oral re-consent from participant
4. Start interview and recording.
5. Completing the IPOS-Dem: “*I would like to ask you to complete the IPOS-Dem (CH) now from the perspective of your relative / client / person with dementia you have most recently cared for. Please speak out loud any thoughts as you continue to complete the IPOS-Dem”.*

- Question 1 (Immediate Reflection) - General: Is there anything urgent you would like to say before we start with the directed questions?
- Question 2-5 can be used interchangeably – go through every item, particular those the participant hesitated to complete:

Question 2: Based on the instructions on the IPOS-Dem, can you tell me in your own words how you completed the IPOS-Dem?

Question 3: Can you please explain to me in your own words what this item is asking about?

- Question 4: Can you please explain to me how you understand the answer options? Was the time period of one week an appropriate time frame?
- Question 5: Did you find it difficult to complete the IPOS-Dem? (Was it difficult to answer the questions? If yes, why? If no, why not)

Prompt: Clarify with participant in detail if an item, phrase or paragraph is difficult to understand and ask to reword the text from his/her point of view.

- Question 6: Is there anything else you would like to add?

1. Stop recording.
2. Thank you to participants / offer support telephone number of study team should any issues arise after the interview.
