# Supplementary material for: Empowering informal caregivers and nurses to take a person-centred view: adaptation and clinical utility of the Integrated Palliative Outcome Scale (IPOS-Dem) for use in acute and community care settings
Source: BMC Geriatr. 2024 Dec 21;24:1030. doi: 10.1186/s12877-024-05608-8 (PMC11662603; doi:10.1186/s12877-024-05608-8)

**Additional file 3 to «Empowering carers and nurses to take a person-centred view: Adaptation and clinical utility of the Integrated Palliative Outcome Scale (IPOS-Dem) for use in acute and community care settings»**

IPOS-Dem (CH) version for the community care setting


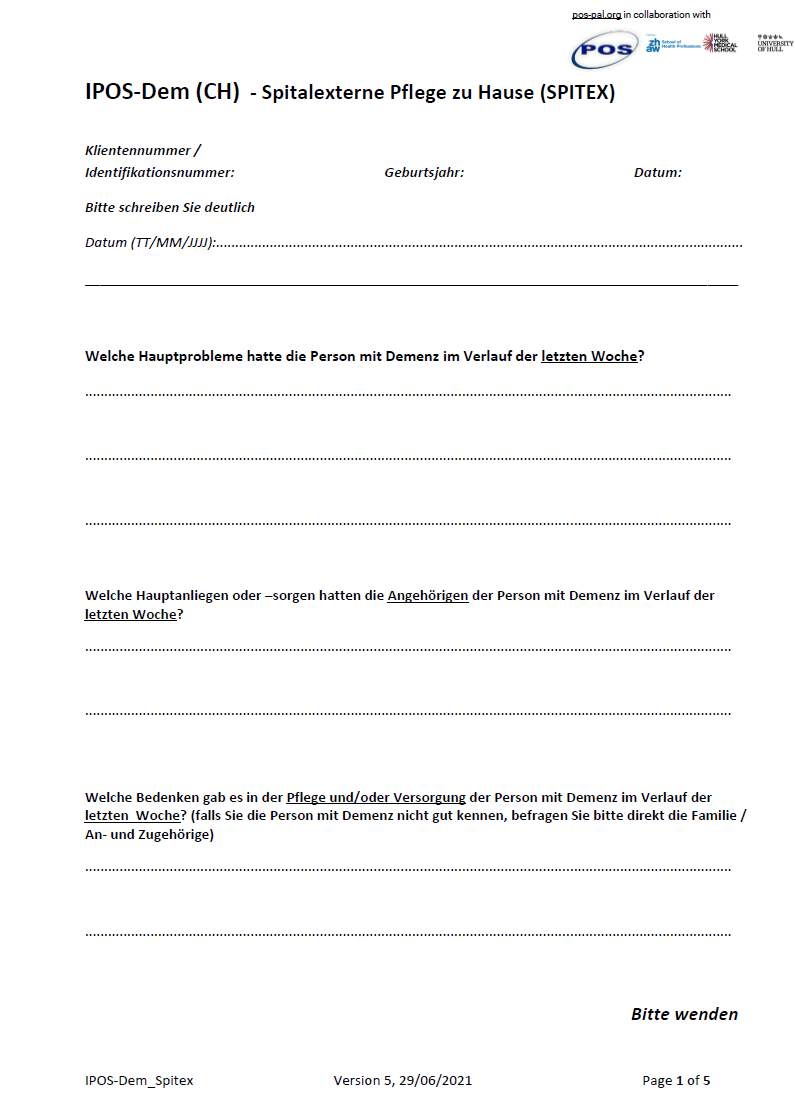


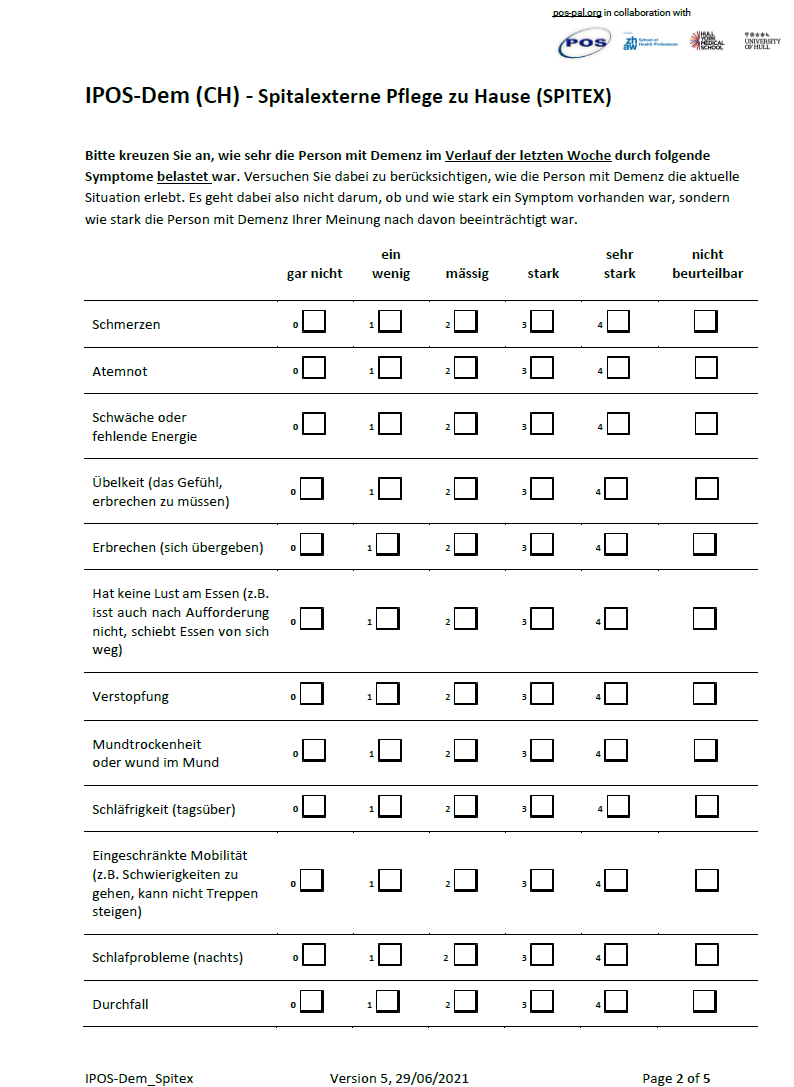


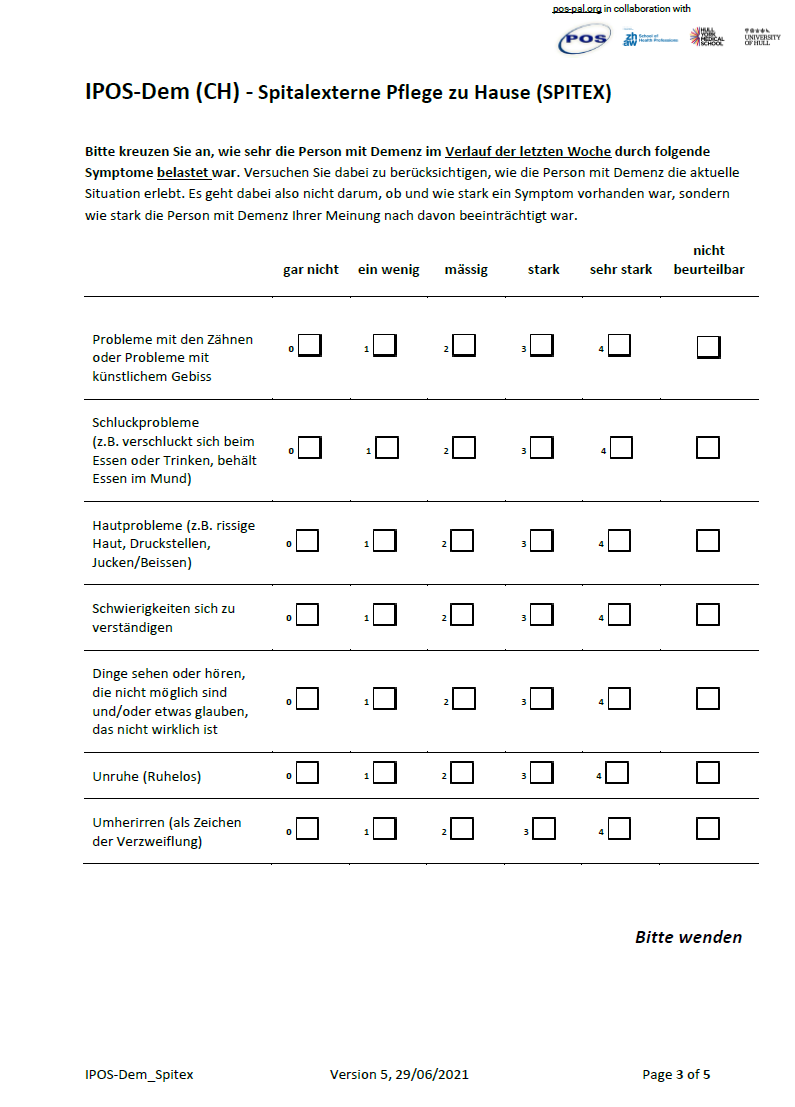


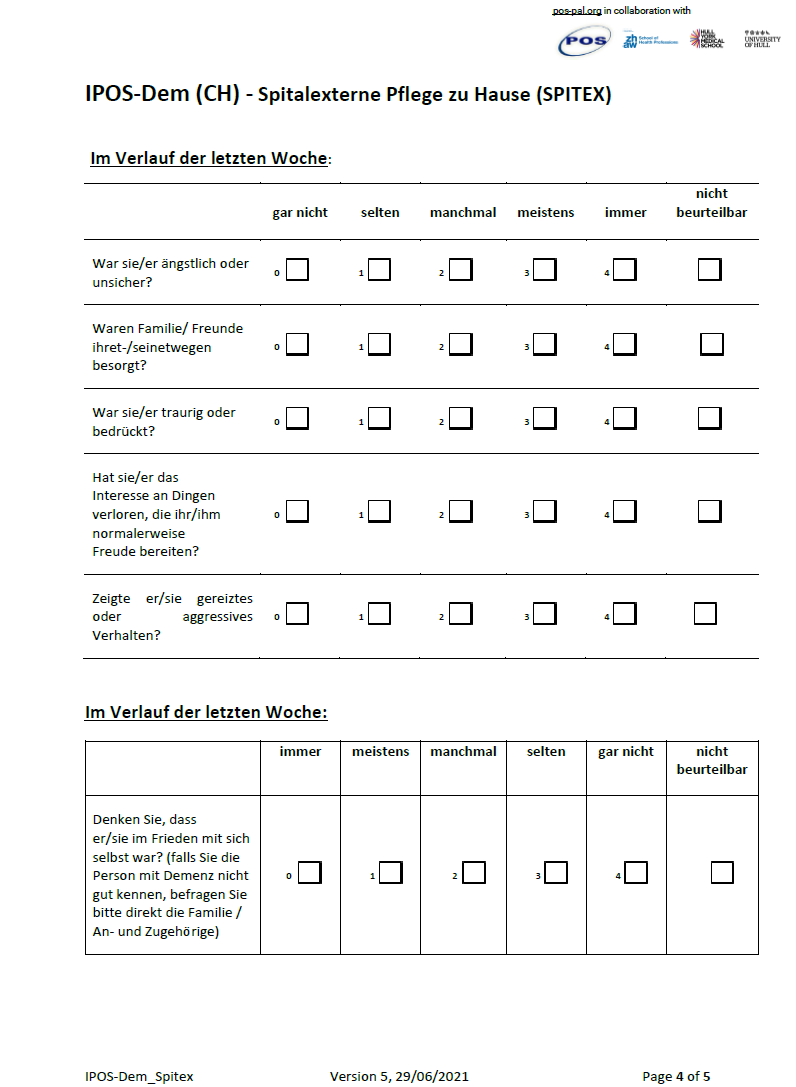


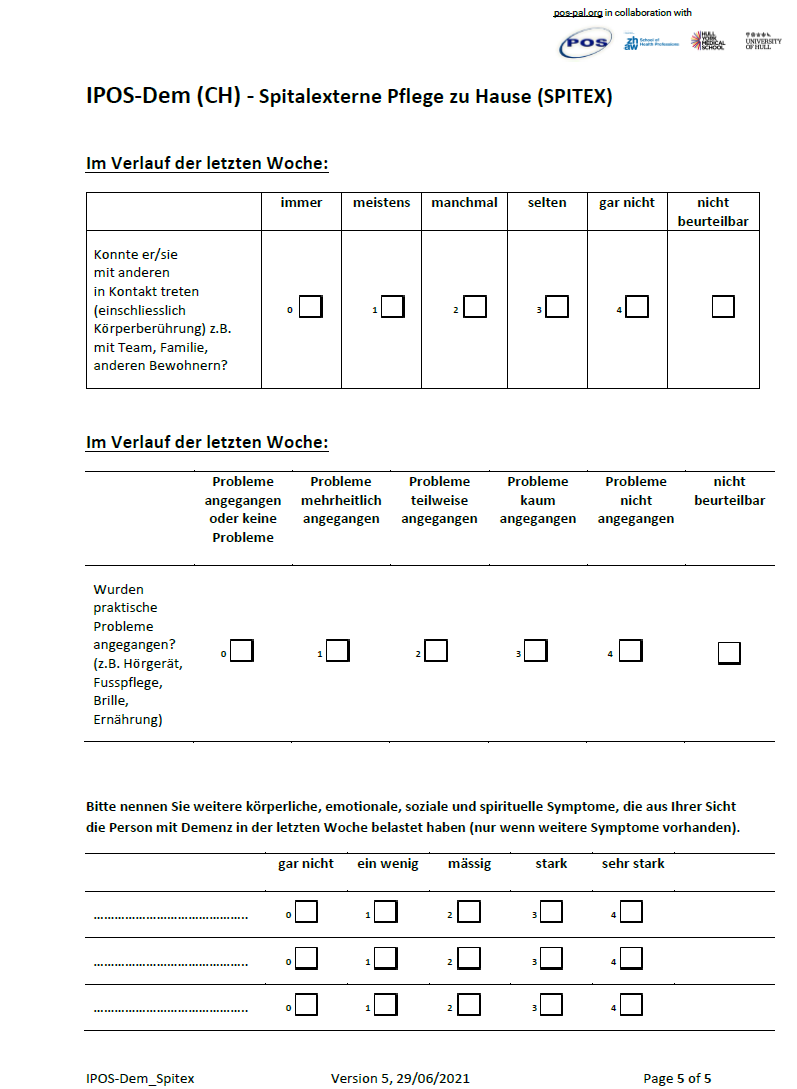


IPOS-Dem (CH) version for the community care setting – back-translation to English


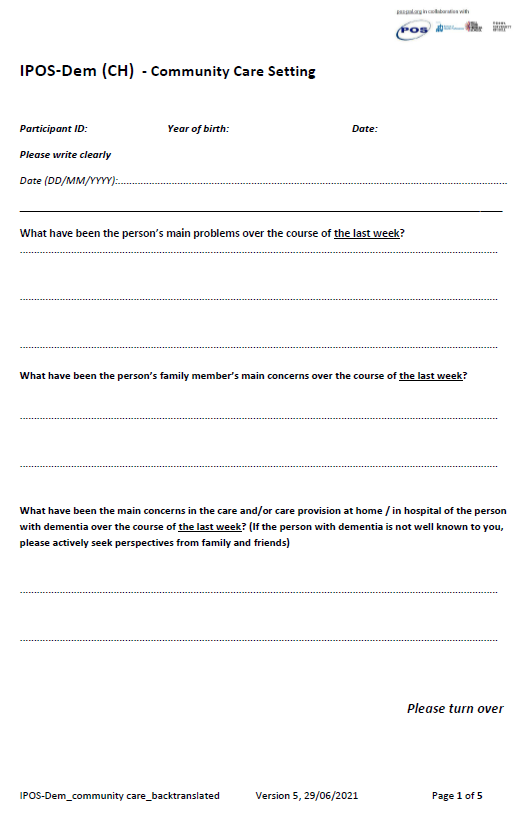


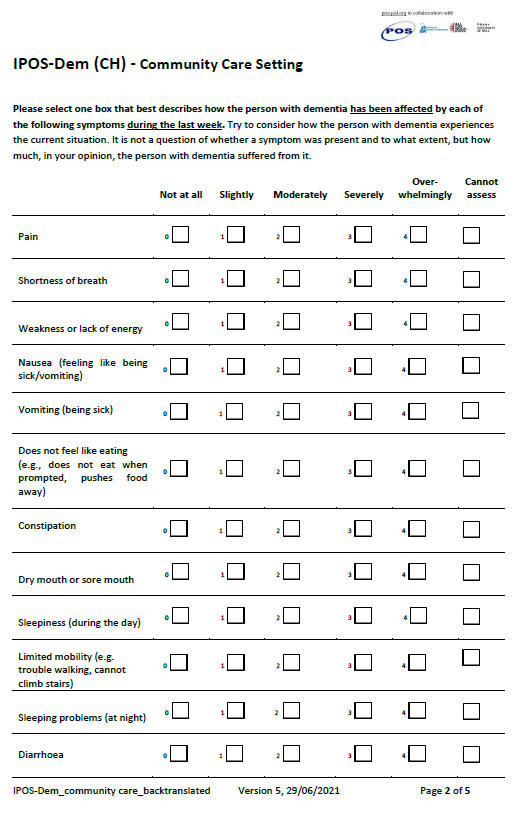


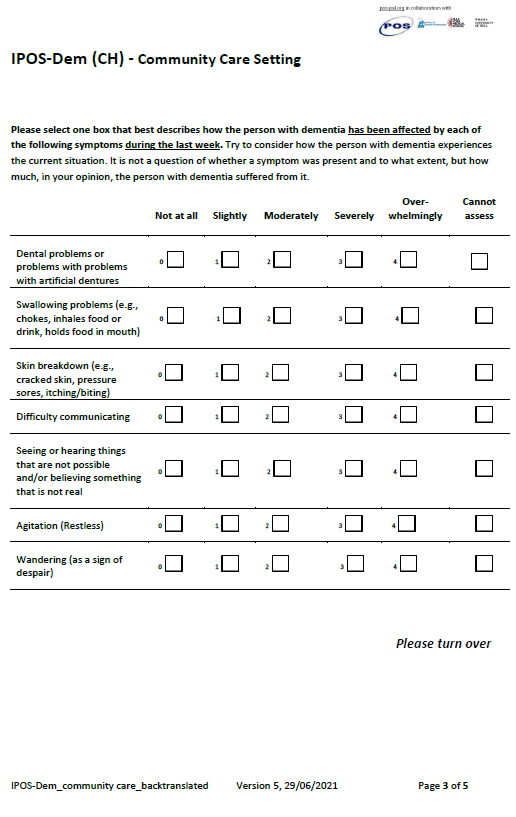


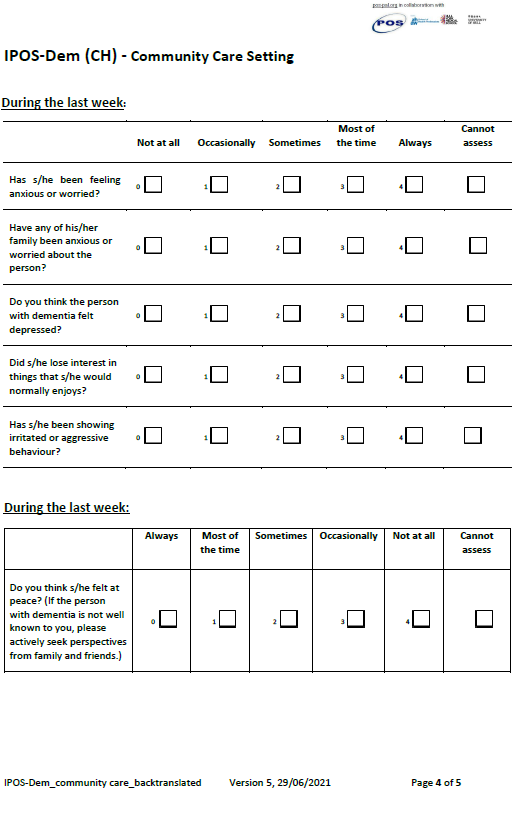


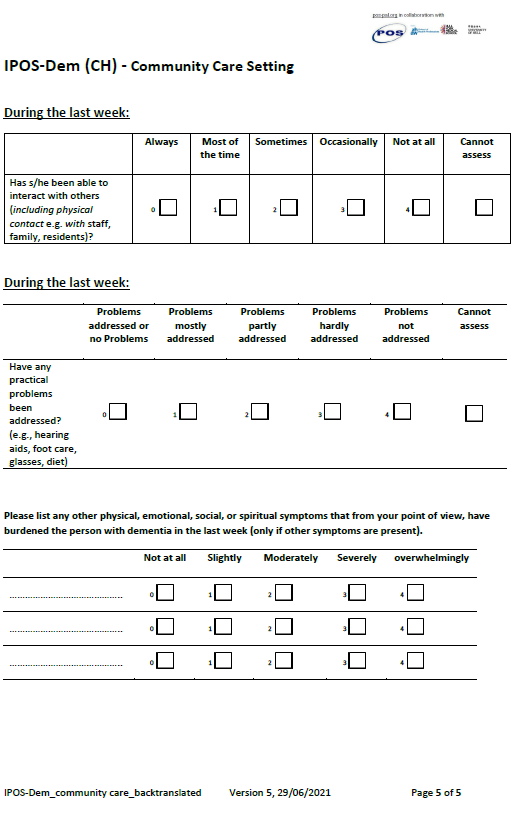

Supplement: Supplementary file 3 — Additional file 3. IPOS-Dem (CH) and back-translated English version for the community care setting. [file 12877_2024_5608_MOESM3_ESM.docx]
